# Case Report: A rare case of gastric-type adenocarcinoma of stumps of duplicated cervices in a 49-year-old woman: A case with hidden focus confused us a lot

**DOI:** 10.3389/fonc.2023.1109731

**Published:** 2023-03-02

**Authors:** Xiaolin You, Li He, Yonghong Lin, Lu Huang, Xihao Wang, Zhigang Wang

**Affiliations:** ^1^ Department of Obstetrics and Gynecology, Chengdu Women's and Children's Central Hospital, School of Medicine, University of Electronic Science and Technology of China, Chengdu, China; ^2^ Department of Pathology, Chengdu Women's and Children's Central Hospital, School of Medicine, University of Electronic Science and Technology of China, Chengdu, China; ^3^ Department of Radiology, Chengdu Women's and Children's Central Hospital, School of Medicine, University of Electronic Science and Technology of China, Chengdu, China

**Keywords:** human papilloma virus, endocervical adenocarcinoma, gastric-type, duplicated cervices, cervical stump carcinoma, didelphic uterus

## Abstract

Gastric-type endocervical adenocarcinoma (G-EAC) is a rare special type of cervical mucinous adenocarcinomas, and it is reported the incidence is unrelated to human papilloma virus infection. We report a rare case of G-EAC in stumps of duplicated cervices in a 49-year-old female patient. The woman complained of post-coital bleeding. She had a didelphic uterus with a duplex cervix, and had undergone subtotal hysterectomy 16 years ago. Gynecological examination revealed a normal-appearing right cervix, but the non-dominant side of the left cervix, which was buried and covered by the side wall of the left vagina, was difficult to view. After exposing, the left side cervix presented a mature appearance which was smaller than a normal cervix. Her serum carbohydrate antigen-19-9 levels was 112.59 U/ml. The right cervix's cytology was normal, whereas the left cervix had unusual glandular epithelial cells. HPV testing on both cervical smears was negative. Adenocarcinoma was identified at 3, 6, 12 o'clock at the right cervix in a colposcopy-directed punch biopsy, while no abnormality was found in the biopsy of the left cervix, nor in the curettage of the double cervices. Pelvic magnetic resonance imaging (MRI) revealed two cervical canals, with a 1.9cm×1.6cm mass inside the left cervix, and the left wall of the right cervix may be involved by the tumor of the left cervix. After much deliberation, we considered that the patient had adenocarcinoma of the left cervix stage IB1. Then, the patient underwent radical cervical resection with bilateral salpingo-oophorectomy and bilateral pelvic lymphadenectomy. Her final histopathology indicated G-EAC of the duplicated cervices. After surgery, she received concurrent chemoradiation. Currently, 29 months after the final chemotherapy was administered, the patient remains healthy. Because G-EAC with duplicated cervices is an uncommon cunning tumor with a bad prognosis, early identification and therapy are recommended to enhance the prognosis. The comprehensive evaluation of symptoms and gynecological examination with cervical cytology, colposcopy-directed punch biopsy, endocervical curettage and MRI examine together may assist in determining an accurate preoperative diagnosis.

## Introduction

Gastric-type endocervical adenocarcinoma (G-EAC) is a rare special type of cervical malignant epithelial tumor. According to the 2014 World Health Organization classification, it is a new variant kind of cervical mucinous adenocarcinoma. G-EAC is the most common type of non-HPV related cervical adenocarcinoma, just second to the usual-type endocervical adenocarcinoma (UEA) associated with HPV 18 infection (1). Minimal deviation adenocarcinoma (MDA) is a highly differentiated subtype of G-EAC. The clinical symptoms are quite atypical. Aqueous secretion and lower abdomen discomfort are two of the most prevalent clinical symptoms. The pathological morphological characteristics are comparable to benign lesions, while the biological characteristics are highly malignant, posing significant diagnostic challenges and affecting the patient’s prognosis. At the moment, initial treatment and postoperative adjuvant therapy are not conclusive, although early diagnosis and treatment are a consensus that can improve prognosis. We present a rare case of gastric-type endocervical adenocarcinoma in stumps of duplicated cervices in a 49-year-old female patient that perplexed us a lot.

## Case report

A 49-year-old woman presented to our hospital in August 2020, complaining of post-coital bleeding with no abnormal vaginal discharging. The patient began having vaginal bleeding after sexual intercourse around 1 month before admission. She had a history of didelphic uterus with cervix duplex. The time of first discovery of her genital malformation was uncertain. And she'd been pregnant four times, with one cesarean section and three induced abortions, including one abnormal pregnancy in the left uterus that resulted in a supracervical hysterectomy around 16 years ago. Previously, she had regular menstrual cycles of 30 days with menstruations of 5-6 days. She had moderate menstruation volume and no dysmenorrhea. The patient had not previously had a routine physical examination. Her latest cervical screening was three years ago, and she underwent an appendectomy eight years ago. There was no family history of tumors or genital malformations in her family.

**Figure 1:**
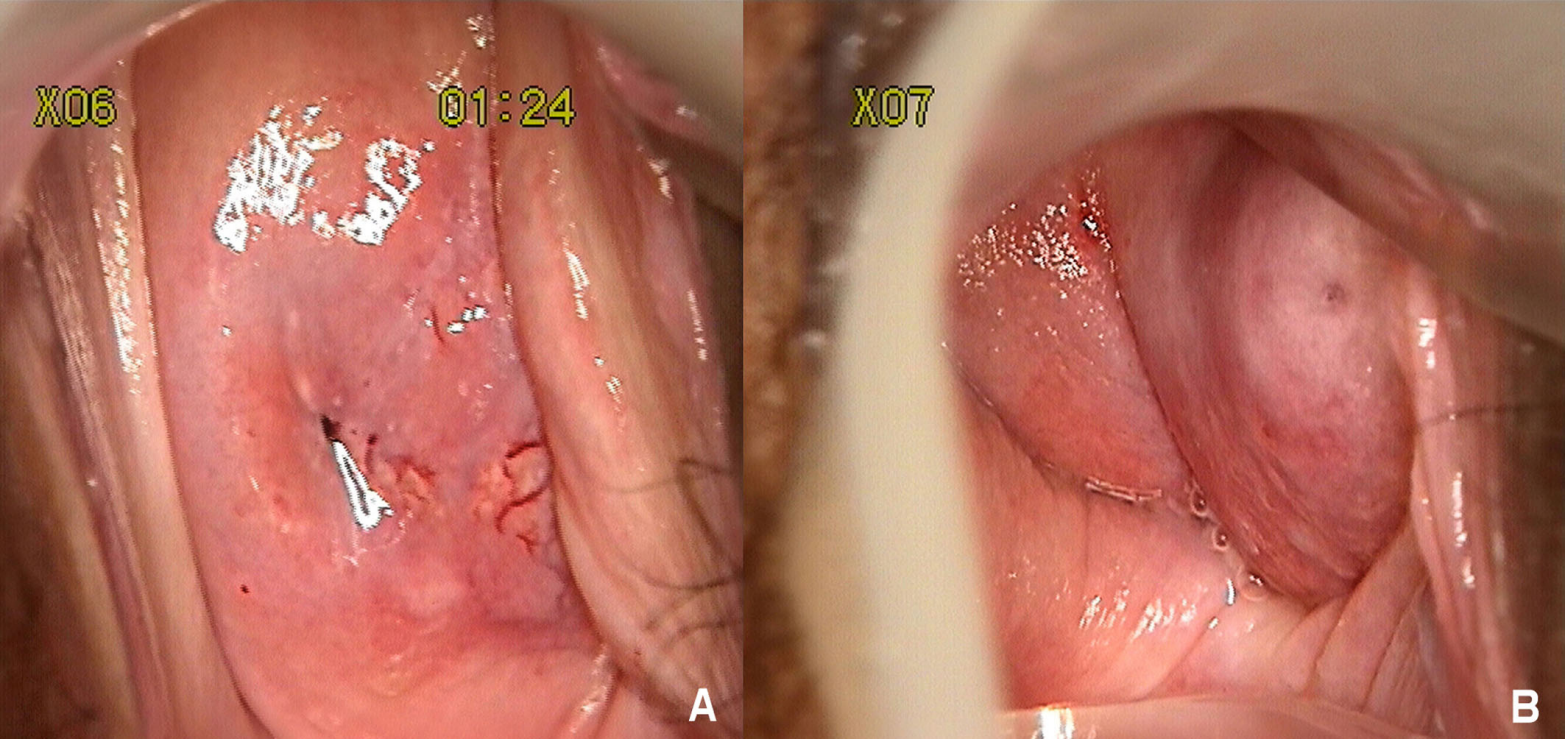
**(A)** Stained with acetic acid, thick vinegar white epithelium, and the accompanying abnormal blood vessels can be seen between 3 and 6 o'clock of the right cervix. **(B)** The nondominant left cervix was concealed by the side wall of the left vagina. After exposing, the left side cervix presented a mature appearance which was smaller than a normal cervix.

Gynecological examination indicated a normal-appearing right cervix, the surface was smooth, but it was easy to bleed when touched, and the non-dominant side of the left cervix was concealed by the side wall of the left vagina. After exposing, the left side cervix presented a mature appearance which was smaller than a normal cervix (**Figure 1**). Bimanual rectovaginal examination can detect the lack of uterine body, pelvic cavity emptiness, and no abnormalities in bilateral parauterine regions.

**Figure 2:**
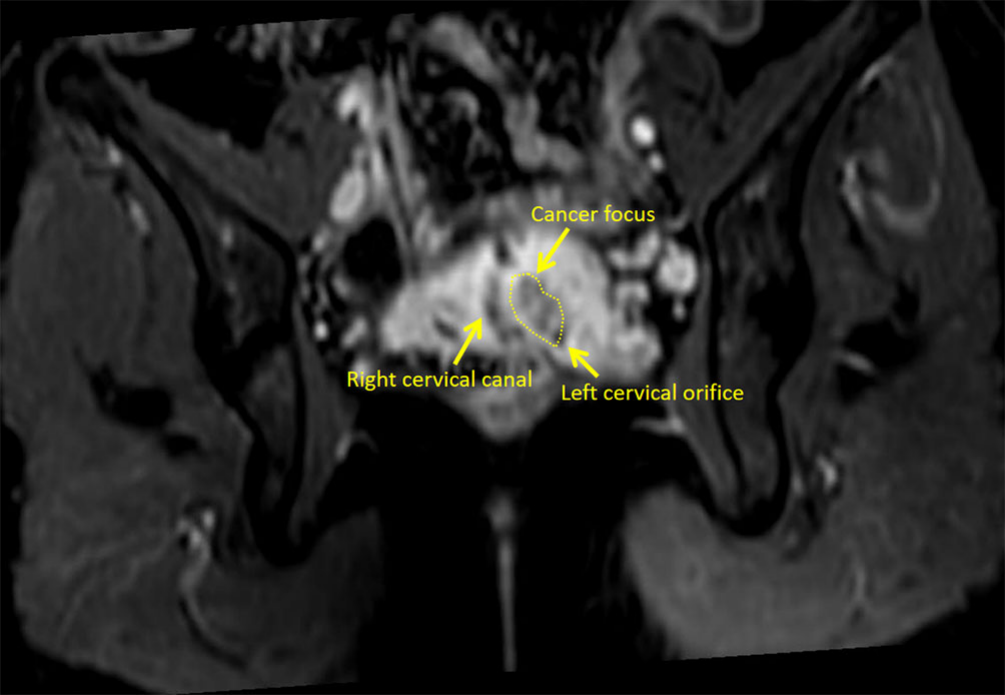
The right cervical canal and the left cervical orifice can be seen in the MRI image at this plane.

Her blood carbohydrate antigen 125 (CA125), carcinoembryonic antigen (CEA), alpha fetoprotein (AFP), and carbohydrate antigen 153 (CA153) levels were all within normal ranges (CA125 17.2 U/ml, CEA < 0.5 ng/ml, AFP 1.5ng/ml, CA153 5.5 U/ml), however the carbohydrate antigen-19-9 (CA19-9) level increased considerably, the corresponding results was 112.59 U/ml. Colonoscopy and gastroscopy revealed no visible abnormalities. The cytology result of liquid-based cell testing in the right cervix was normal, whereas the left cervix revealed atypical glandular epithelial cells. Both cervical smears were negative for human papilloma virus (HPV), including high risk HPV16, 18, 31, 33, 35, 39, 45, 51, 52, 53, 56, 58, 59, 66, 68, 73, 82, and low risk HPV6, 11, 42, 43, 81, 83. Histopathology of the two cervices from colposcopy-directed punch biopsy (CDB) and endocervical curettage (ECC) revealed: 1. (RIGHT CDB) adenocarcinoma at 3,6,12 o'clock on the right cervix, IHC: ER (focal weak+), CK7 (+), CDX2 (-), Muc-6 (-), P53 (+, positive rate of 20%), Ki67 (approximately 30%); 2. (RIGHT ECC) the submitted tissue is a small blood clot; 3. (LEFT ECC) the submitted tissue is a little mucus; 4. (LEFT CDB) chronic inflammation at the left cervix. Pelvic magnetic resonance imaging (MRI) revealed two cervical canals and a 1.9cm×1.6cm mass inside the left cervix (**Figure 2**). The left wall of the right cervix may be involved by the tumor in the left cervix. A few small lymph nodes on both sides of the pelvic could be seen. And there was no lymphovascular space invasion or enlarged pelvic lymph node identified. In addition, there was only one right kidney, with no left kidney.

The biopsy of the patient's right cervix indicated adenocarcinoma, while MRI revealed that the tumor was situated in the left cervical canal and had invaded the neighboring right cervix. After much deliberation, the diagnosis of adenocarcinoma of the left cervix stage [IB1, International Federation of Gynecology and Obstetrics (FIGO), (2)] and stage T1b1N0M0 [American Joint Committee on Cancer (AJCC), (3)] was made. In the first step, we performed cystoscopy, the right ureteral D-J tube implantation, and pelvic sentinel lymph node biopsy under laparoscope. The hyperstaging of pathology revealed that three sentinel lymph nodes had no cancer metastases. Then, we conducted radical cervical resection with bilateral salpingo-oophorectomy, and bilateral pelvic lymphadenectomy without the use of a manipulator. The intraoperative diagnosis was consistent with the preoperative diagnosis. The double cervices were dissected after surgery.

**Figure 3:**
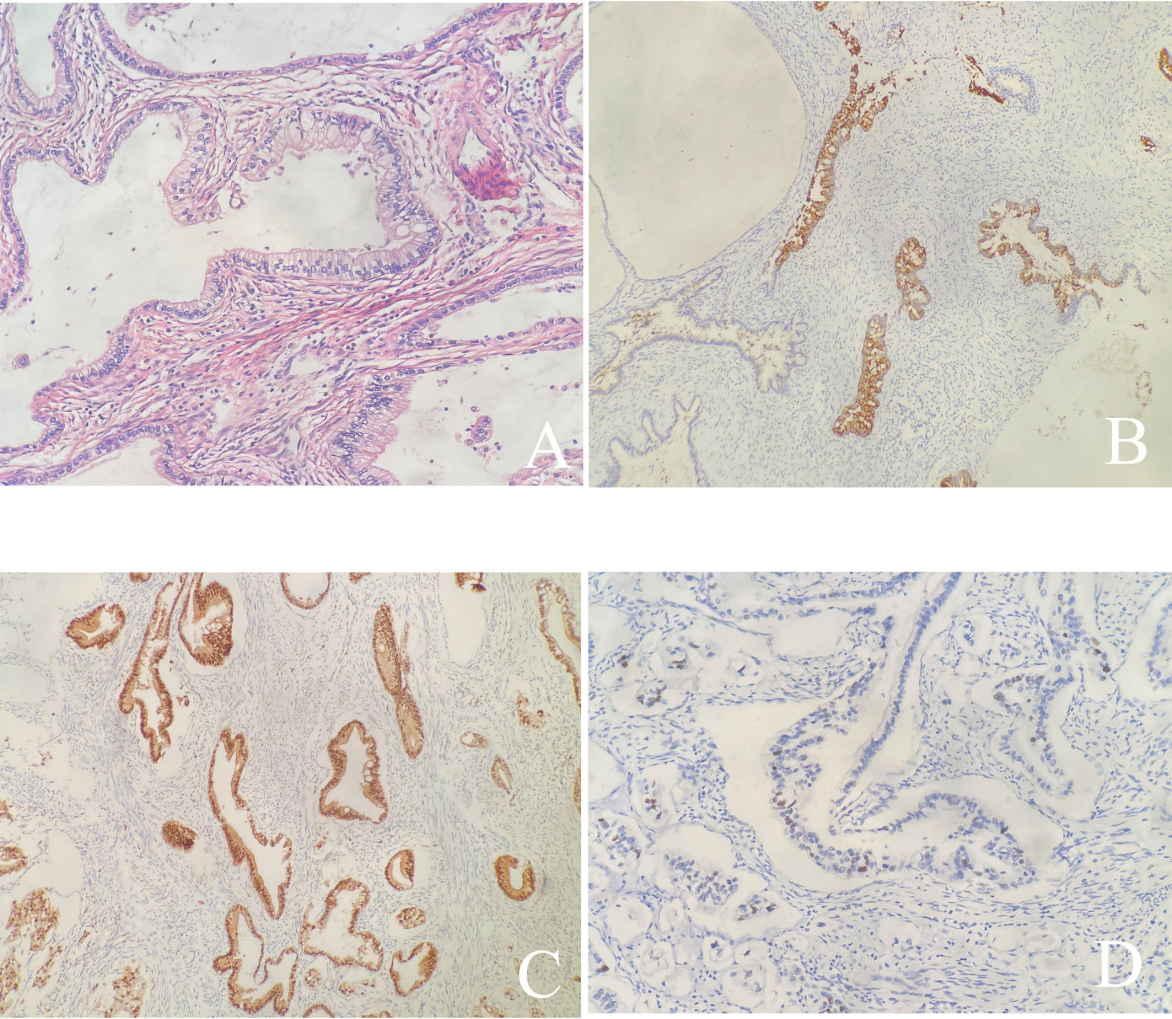
**(A)** Hematoxylin and Eosin stain (×200): The tumor shows gastric type differentiation with abundant clear or pale, eosinophilic cytoplasm with atypical nucleus; **(B-D)** Immunohistochemical stains are focally positive for Muc-6 **(B)**, P53 **(D)**, and positive for P16 **(C)**.

After the resection, the morphology of the double cervices was seen to be irregular. Tumors were seen between the duplex cervices' circumferential walls. The tumor was around 2cm×2cm×2cm in the left cervix and penetrated the neighboring right cervical tissue without infiltrating the vagina or the parametrium. Microscopically, the morphology and immunohistochemistry of the duplicated cervices revealed gastric-type adenocarcinoma with high and intermediate differentiation, as well as intestinal differentiation in some regions. The tumor grew endogenously, penetrating the whole layer of the left cervical wall as well as the shallow layer of the right cervical wall (about 1/3 of the cervical wall). There was lymphatic vascular space infiltration (LVSI), but no clear nerve invasion or malignancy in the bilateral parauterine, vaginal stump, or vaginal vault. Pathological examination revealed no tumor involvement in the lymph nodes. There was no evidence of malignancy in the ovarian and fallopian tube tissues on either side. Additionally, no cancer metastases was discovered in the lymph nodes that were removed (0/18). Immunohistochemistry of the tumor cells were as follows: Muc-6 (+, focally), P16 (+, focally), P53 (20% medium intensity +), ER (+, focally), PR (-), CK7 (+++), CK20 (+, focally), Muc-2 (+, focally), Muc-5 (++), CDX-2 (+), SATB2 (-), Pax-8 (-), and the ki67 proliferative index was around 15% (**Figure 3**). All 23 HPV subtypes tested negative, including high-risk HPV16, 18, 31, 33, 35, 39, 45, 51, 52, 53, 56, 58, 59, 66, 68, 73, 82, and low-risk HPV6, 11, 42, 43, 81, 83. Following surgery, she had concomitant chemoradiation, a total dosage of 60 Gy pelvic irradiation, and three cycles of chemotherapy comprising paclitaxel and carboplatin. Currently, 29 months after the final treatment, the patient has normal cytology and colposcopy results with negative HPV tests.

## Discussion

Gastric-type endocervical adenocarcinoma (G-EAC) is a more heterogeneous group of tumors that is not linked to HPV infection. In 2014, the WHO initially introduced the G-EAC disease category based on its morphological and cellular characteristics (4). G-EAC is poorly symptomatic and very aggressive, usually with myometrial and even serosa invasion, parametrium infiltration, and lymphatic vascular space infiltration (LVSI). In 2018, the International Endocervical Adenocarcinoma Criteria and Classification (IECC) scheme was proposed, which categorized cervical adenocarcinomas into HPV-associated and HPV-independent types (5–7). Gastric-type, mesonephric, and clear cell adenocarcinomas are HPV-independent types of cervical adenocarcinoma (7), in which G-EAC is the most common kind. G-EAC is estimated to account for 10% of all cervical adenocarcinomas in European and American countries (5). In China, it affects about 16% of the adenocarcinomas of the cervix (8).

Only a few cases of cervical neoplasms in people with a duplex cervices have previously been reported (9). Duplication of the genital tract may make routine pathology diagnostically more challenging. Endocervicoscopy, which coupled traditional hysteroscopic apparatus with the colposcopic categorization system, was reported to aid evaluation of the endocervical mucosa in a subset of patients when colposcopy was insufficient (10). Our patient had a didelphic uterus with a duplex cervix, and had undergone subtotal hysterectomy 16 years ago. The appearance of her right cervix was normal, cytological examination indicated no abnormalities, colposcopy revealed squamous intraepithelial lesions in the right cervix, and the biopsy indicated adenocarcinoma in the right cervix. The appearance of her left cervix was slightly smaller than the normal one, the cytological examination revealed atypical glandular epithelial cells, and the cervical endocervical curettage and biopsy found no abnormalities. MRI showed that the mass was mainly inside the left cervix. The lesion site appeared to be inconsistent with the examination results, which perplexed us initially. After much deliberation, we considered that the tumor was primarily located in the left cervix, exhibiting endogenous growth and infiltrating the neighboring right cervical wall. Finally, the diagnosis of cervical cancer stage IB1 (2) and stage T1b1N0M0 (3) in the left cervix was made.

The clinical signs of G-EAC is lack of specificity. Increased vaginal discharge, or lower abdominal pain may be the first symptom in some patients, but contact bleeding and irregular vaginal bleeding are uncommon. Gynecological examinations usually reveal the external opening of the cervices being generally smooth or erosive, and the lesions being mostly buried in the cervical tube. The detection of high-risk HPV is typically negative. And the cytological detection rate is low, due to the atypical changes in cytological characteristics, the difficulty in obtaining specimen, and even to the insufficient understanding of pathologists. More than half of G-EAC patients have increased CA199 levels, and nearly one-third have elevated CA125 levels, while the increasement of CA125 usually indicates the presence of pelvic and peritoneal metastasis (11, 12). MRI, on the other hand, is a good and accessible diagnostic technology that can explain the features of lesions and evaluate local regional expansion. The characteristic of G-EAC in MRI is classified as a "cosmic model": the lesion is generally placed at a higher location of the cervix, and there are some tiny cysts or solid components in the core of the lesion, surrounded by relatively big cysts (13, 14).

The IECC describes G-EAC as tumor cells with extensive translucent, foamy, or light eosinophilic cytoplasm. On the whole, the proportion of cell nucleus to cytoplasm is low, and tumor cells are disorganizedly scattered in the gland's basal region. Kojima et al. proposed three morphological criterias: abundant mucinous cytoplasm, translucent or light eosinophilic cytoplasm, and a distinct cell border (15). In 2019, Pirog et al. expanded the morphological description of G-EAC (16). The nucleus of the gland cells are slightly to moderately heterotypic, with small nucleoli, less pathological mitosis and necrosis. And varying degrees of fibrogenic interstitial response can be seen in the interstitium around the nucleus. The differentiated glands composed by smaller cuboidal or flat cells expand into papillary single tubes and are dispersed throughout the stroma. Goblet cells with thick eosinophilic and visible foam-like cytoplasm are scattered in the gland. MUC6 and HIK1083 are two conventional immunohistochemical markers of gastric mucus. P16 is commonly negative or focally positive in G-EAC, while it is diffusely positive in HPV-associated usual-type endocervical adenocarcinoma (UEA). However, it has been shown that P16 may be diffusely strong positive in some G-EACs. P53 mutations were found in 50% of patients (17). Mutant p53 expression may be the primary molecular mechanism of carcinogenesis, as well as the tumor's resistance to radiation and chemotherapy. CK7, CDX2, PAX8, CA199, and CEA are all mostly focal or diffusely positive. The paired nuclear gene 2 protein (PAX2) and the estrogen and progesterone receptors (ER, PR) are generally negative (18), indicating that the tumor may not be connected to the patient's hormone level. Furthermore, the Ki 67 proliferation index is frequently low, leading to the illusion that the tumor's biological activity is relatively benign. In general, G-EAC is difficult to be identified, the missed diagnostic rate is as high as 34% (19). The pathological morphology looks like "benign", while the biological behavior is extremely aggressive. Routine cytological examination, even with colposcopy-directed punch biopsy, endocervical curettage, cervical scratch, or other histological investigations, is difficult to detect the presence of lesions. Deeper tissues are frequently required to provide a good diagnosis, even with conization tissue of the cervix when necessary. Nakamura et al. examined 322 instances of cervical cancer and discovered that preoperative biopsy is difficult to identify G-EAC, but some special clinical signs, such as aqueous secretion and lower abdominal discomfort, high serum CA19-9 levels, and immunohistochemistry of HIK1083 and MUC6 staining can aid in the diagnosis (11).

Common gastric metaplasia, lobular cervical hyperplasia, tunnel-like glandular plexus, atypical phylloid cervical endometrial hyperplasia (LEGH), and cervical gastric adenocarcinoma *in situ* (AIS) are the precursor lesions of G-EAC. When cervical gastric mucinous lesions exit, we should be aware of the existence of gastric mucinous lesions in other sections of the body at the same time, i.e., synchronous mucinous metaplasia and neoplasia of the female reproductive tract (SMMN-FGT). SMMN-FGT, also known as multifocal gastric mucinous lesions, is the term used to describe the simultaneous development of gastric mucinous lesions in two or more areas of the female reproductive system. Additionally, according to the literature, Peutz-Jeghers Syndrome (PJS) may be associated to G-EAC (20, 21). PJS typically presents with polyps in the digestive tract along with oral, labial, buccal mucosa, and plantar black patches. About 30%~50% of the patients had autosomal dominant inheritance, and STK11 was its pathogenic gene. Patients with PJS are advised to undergo routine annual physical exams, and patients with G-EAC are advised to obtain genetic counseling and look for pertinent molecular biological markers.

The most prevalent mutant gene of G-EAC was found to be TP53, followed by STK11, HLA-B, PTPRS, FGFR4, GNAS, BRCA2, and others (1). It was reported that human epidermal growth factor receptor-2 (HER-2) amplification in G-EAC is more frequent than in other cervical adenocarcinomas, and it is more prevalent in individuals with ovarian metastases and late stage (22). Garg et al. used next-generation sequencing (NGS) to examine 161 distinct cancer-driver genes to clarify the molecular features of gastric cervical adenocarcinoma (23). They discovered that there may be certain molecular abnormalities that are possibly operable and demonstrated the genetic heterogeneity of gastric cervical cancer. Lu et al. employed NGS technology to discover specific genomic alterations of G-EAC, and revealed indicate that the genomic alterations mainly involved the cell cycle and PI3K/AKT signaling pathways (24). Toll-like receptors (TLR) 2 (196 to 174 del) and TLR 4 (Asp299Gly, Thr399Ile) were reported to be involved with cervical cancer susceptibility (25). It is worth investigating whether TLR polymorphisms are associated with G-EACs.

As the biological behavior features of G-EAC are similar to those of small cell neuroendocrine carcinoma of the cervix, the Chinese Expert Consensus on the Clinical Diagnosis and Treatment of Cervical and Gastric Adenocarcinoma (2021 Edition) (19) indicated that in the absence of standard treatment approaches, the treatment of G-EAC can refer to the 2021 NCCN Clinical Practice Guidelines for cervical small cell neuroendocrine cancer (First Edition) . This guideline points out that for patients with tumors ≤ 4 cm, radical hysterectomy+pelvic lymphadenectomy ± para-aortic lymph node sampling is the first choice for surgery (26). In view of the high invasiveness of G-EAC, retaining fertility and ovary is not advised. For G-EAC’s propensity of metastasis and accompanying SMMN-FGT, the appendix and omentum majus can be removed in operation. Surgery for patients with ovarian metastases should be comparable to tumor cytoreductive surgery. The postoperative adjuvant therapy approaches are chosen based on the intraoperative circumstances and postoperative pathology diagnosis. Postoperative adjuvant therapy is advised if any of the high risk factors exist: positive lymph node, positive surgical margin, and periuterine infiltrate. The histological type of adenocarcinoma is also one of the medium risk factors according to the sedlis standard (26).

With the propensity of infiltrating neurons and blood vessels, high expression rate of P53 mutation, and resistance to chemotherapy and radiation, the prognosis of G-EAC is worse than those of UEA and squamous cell carcinoma (27–29). The recurrence rate of G-EAC is roughly 40%, and the 5-year survival rate is only about 32% (15, 29). According to a research, patients with G-EAC that is positive for the programmed death ligand 1 (PD-L1) have a poorer prognosis than those with negative PD-L1 in terms of both disease progression-free survival and overall survival (30).

Following the publication of the LACC study, minimally invasive surgery for cervical cancer became controversial (31). The most recent NCCN recommendation (2022) maintains the minimally invasive method as a treatment option for radical trachelectomy. Our patient was diagnosed with cervical cancer stage IB1 (2) in the left cervix preoperatively. After being fully informed about the outcomes and oncologic risks associated with various surgical approaches, the patient underwent laparoscopic radical cervical resection with bilateral salpingo-oophorectomy and lymphadenectomy without the use of a uterine manipulator, as well as immediate bagging to prevent tumor spillage. Her final histology revealed that she had gastric-type adenocarcinoma of the double cervical stumps. After surgery, she received concurrent chemoradiation. Currently, 29 months after the final chemotherapy was administered, the patient remains healthy. However, the patients did not receive genetic testing. It is unknown whether a genetic factor contributed to the abnormality. Furthermore, the present case is not sufficient to conclude the management of G-EACs of cervical lesion on both cervices. More data is needed to establish the management of cervical lesion on both cervices based on the current instance.

Cervical cancer in individuals with duplicated cervices is rare; nonetheless, we provide a novel case of gastric-type endocervical adenocarcinoma in stumps of duplicated cervices. There are certain limitations, though. Since the individuals did not undergo genetic testing, it was not possible to identify whether a hereditary component contributed to the deformity and the cervical lesion. In addition, the patient's prior supracervical hysterectomy, performed around 16 years ago, was not performed in our facility, and the specific circumstances were unknown. More evidence was necessary since the current instance was insufficient to draw any conclusions on the management of the cervical lesions on both cervices.

## Conclusion

Gastric-type endocervical adenocarcinoma in stumps of duplicated cervices is kind of rare cunning tumor with poor prognosis that is difficult to detect in cervical precancer screening. Early detection and timely treatment of this cervical precancerous lesions are very important for its treatment. The comprehensive evaluation of symptoms, gynecological examination, cervical cytology, colposcopy-directed punch biopsy, endocervical curettage, and MRI scan may all aid in determining a precise preoperative diagnosis. And molecular characterization guided to G-EAC’s targeted therapy need to be further identified.

## Data availability statement

The original contributions presented in the study are included in the article/supplementary material. Further inquiries can be directed to the corresponding author.

## Ethics statement

Written informed consent was obtained from the individual(s) for the publication of any potentially identifiable images or data included in this article.

## Author contributions

XY, LHe, and YL worked on the case and wrote the manuscript. XY, LHu, XW, and ZW participated in the collection of case data and literature, as well as the completion of all documentary and article work. LHe and YL has given many constructive suggestions for this paper. All authors contributed to the article and approved the submitted version.
